# A Case Report of Sequential Use of a Yeast-CEA Therapeutic Cancer Vaccine and Anti-PD-L1 Inhibitor in Metastatic Medullary Thyroid Cancer

**DOI:** 10.3389/fendo.2020.00490

**Published:** 2020-08-07

**Authors:** Jaydira Del Rivero, Renee N. Donahue, Jennifer L. Marté, Ann W. Gramza, Marijo Bilusic, Myrna Rauckhorst, Lisa Cordes, Maria J. Merino, William L. Dahut, Jeffrey Schlom, James L. Gulley, Ravi A. Madan

**Affiliations:** ^1^Genitourinary Malignancies Branch, Center for Cancer Research, National Cancer Institute, Bethesda, MD, United States; ^2^Developmental Therapeutics Branch, Center for Cancer Research, National Cancer Institute, Bethesda, MD, United States; ^3^Laboratory of Tumor Immunology and Biology, Center for Cancer Research, National Cancer Institute, Bethesda, MD, United States; ^4^Medstar Georgetown Lombardi Comprehensive Cancer Center, Georgetown University Medical Center, Washington, DC, United States; ^5^Laboratory of Pathology, National Cancer Institute, Bethesda, MD, United States

**Keywords:** medullary thyroid cancer, CEA, calcitonin, immunotherapy, PD-L1 inhibitor

## Abstract

Medullary thyroid cancer (MTC) accounts for ~4% of all thyroid malignancies. MTC derives from the neural crest and secretes calcitonin (CTN) and carcinoembryonic antigen (CEA). Unlike differentiated thyroid cancer, MTC does not uptake iodine and I-131 RAI (radioactive iodine) treatment is ineffective. Patients with metastatic disease are candidates for FDA-approved agents with either vandetanib or cabozantinib; however, adverse effects limit their use. There are ongoing trials exploring the role of less toxic immunotherapies in patients with MTC. We present a 61-year-old male with the diagnosis of MTC and persistent local recurrence despite multiple surgeries. He was started on sunitinib, but ultimately its use was limited by toxicity. He then presented to the National Cancer Institute (NCI) and was enrolled on a clinical trial with heat-killed yeast-CEA vaccine (NCT01856920) and his calcitonin doubling time improved in 3 months. He then came off vaccine for elective surgery. After surgery, his calcitonin was rising and he enrolled on a phase I trial of avelumab, a programmed death-ligand 1 (PD-L1) inhibitor (NCT01772004). Thereafter, his calcitonin decreased > 40% on 5 consecutive evaluations. His tumor was subsequently found to express PD-L1. CEA-specific T cells were increased following vaccination, and a number of potential immune-enhancing changes were noted in the peripheral immunome over the course of sequential immunotherapy treatment. Although calcitonin declines do not always directly correlate with clinical responses, this response is noteworthy and highlights the potential for immunotherapy or sequential immunotherapy in metastatic or unresectable MTC.

## Introduction

Medullary thyroid cancer (MTC) accounts for ~4% of all thyroid malignancies. It is a neuroendocrine tumor deriving from the neural crest-derived parafollicular or C cells of the thyroid gland (1). About 75% of MTC cases are sporadic and the remaining 25% present as part of an autosomal dominant inherited disorder. Activating mutations of the *RET* (Rearranged during Transfection) proto-oncogene are characteristic, with germline activating RET mutations seen in fMTC (familial MTC) and MEN (multiple endocrine neoplasia) 2a/MEN2b (2–4). MTC most often produces both immunoreactive calcitonin (CTN) and carcinoembryonic antigen (CEA), which are used as tumor markers (5). The growth rate of MTC is estimated by using RECIST v.1.1 (Response Evaluation Criteria in Solid Tumors); however, it can also be determined by measuring serum levels of CTN and CEA over multiple time points to determine doubling time, which play an important role in the follow-up and management of MTC. Calcitonin doubling times of >2 years seem to be associated with a better long-term prognosis than those of <6 months (6, 7).

The role of immunotherapy in MTC is not fully studied. However, previous studies have identified evidence of T-cell infiltration on MTC (8). Dendritic cell (DC)-based immunotherapy was also given in patients with solid tumors including MTC and it was reported that vaccination with autologous tumor-pulsed DCs generated from peripheral blood was safe and can induce tumor-specific cellular cytotoxicity (9). Schott et al. (10) reported that subcutaneous injection of calcitonin and CEA loaded DC vaccine in patients with metastatic medullary thyroid cancer showed clinical benefit. Calcitonin and CEA decreased in 3 of 7 patients and one of these patients had complete regression of detectable liver metastasis and reduction of pulmonary lesions. A phase I study using the heat-killed yeast-CEA vaccine (GI-6207) was performed at the National Cancer Institute (NCI) (11). A total of 25 patients were enrolled in a classic phase I design at 3 dose levels. One patient with MTC had a significant inflammatory response at the sites of her tumors and a substantial and sustained antigen-specific immune response. Furthermore, the relatively low toxicity profile of therapeutic cancer vaccines could be advantageous compared to approved tyrosine kinase inhibitors (TKIs) for some patients with indolent recurrent or metastatic MTC. Here we present a case of a patient with recurrent MTC who was enrolled on a clinical trial with yeast-based vaccine targeting CEA. Upon surgical resection after vaccine, his tumor was found to express programmed death-ligand 1 (PD-L1), which may explain the patient's subsequent reponse to a PD-L1 inhibitor.

## Case Presentation

We report a 61-year-old male who initially presented with an enlarging anterior neck mass that was biopsied and found to be consistent with the diagnosis of MTC (no known somatic or germline mutation of the *RET* proto-oncogene). Subsequently, he underwent a total thyroidectomy with bilateral neck lymph node dissection. He then had multiple local recurrences, resulting in a total of five neck surgeries, the last one occurring 12 years after diagnosis. Based on the elevated CTN levels and persistent local recurrence, he then started systemic treatment with off-label sunitinib 13 years after diagnosis (12). While on sunitinib his CTN levels nadired to 199 pg/ml (reference <10 pg/ml), down from 461 pg/ml 6 months after starting treatment. He continued for 5 years and then stopped due to side effects. His CTN levels after discontinuing sunitinib rose to 2,243 pg/ml.

On follow-up imaging studies, there was no evidence of distant metastases and he presented to the NCI with disease involving his thyroid bed and cervical nodes, most of which were not amenable to resection. He then enrolled on a clinical trial with yeast-based therapeutic cancer vaccine targeting CEA (a phase 2 study of GI-6207 in patients with recurrent medullary thyroid cancer; NCT01856920) (8, 13). During a 6-month protocol–mandated surveillance, he had a CTN of 2,225 pg/mL and CEA of 11.9 ng/mL (reference 0.8–3.4 ng/mL) that increased to 5,964 pg/mL and CEA of 20.6 ng/mL (CTN doubling time of 135 days). During the subsequent 3-month vaccine period, his doubling time improved to 530 days (nadir CTN was 4,503 pg/mL and CEA 19 ng/mL). He then chose to have elective surgery to remove a neck lymph node and, per protocol, the vaccine was discontinued.

**Figure 1:**
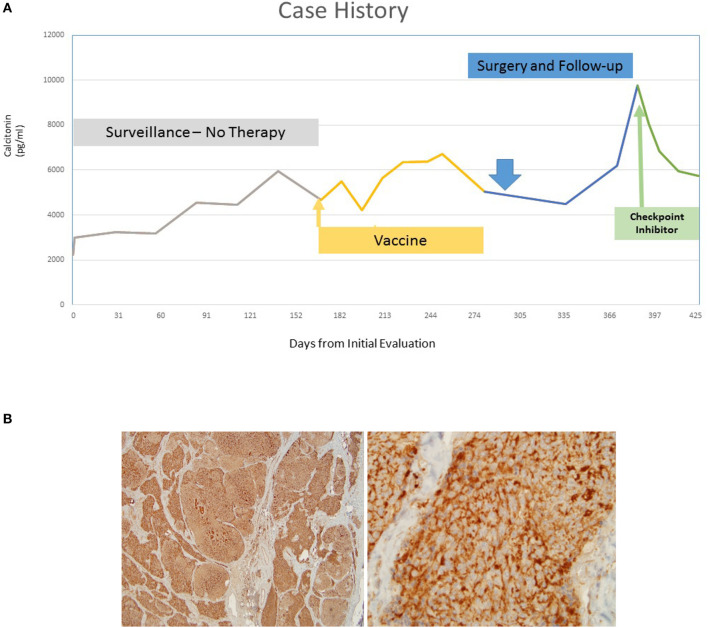
**(A)** Five consecutive declines in the patient's calcitonin levels while on the immune checkpoint inhibitor, a > 40% decline. **(B)** Robustly positive PD-L1 staining after surgical resection of a neck lymph node after vaccine (higher power on the right).

**Figure 2:**
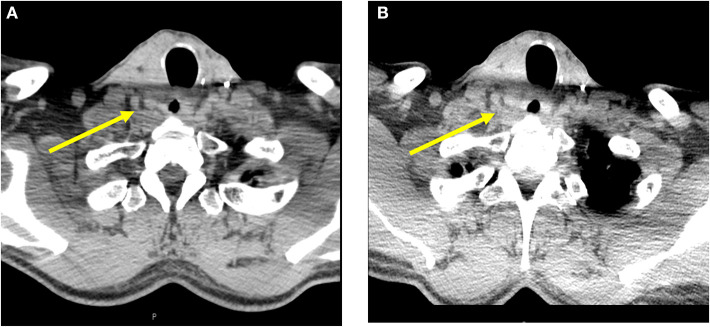
Cross sectional imaging studies with computed tomography of the neck **(A)** prior to PD-L1 administration and **(B)** after a 40% decrease in calcitonin, showing stable thyroid bed recurrence.

Approximately 3 months after surgery, his calcitonin had risen to 9,765 pg/ml and CEA 17.1 ng/mL and the patient was enrolled on a phase I trial of avelumab, a PD-L1 inhibitor (phase I, open-label, multiple-ascending dose trial to investigate the safety, tolerability, pharmacokinetics, biological, and clinical activity of avelumab (MSB0010718C), a monoclonal anti-PD-L1 antibody, in subjects with metastatic or locally advanced solid tumors (NCT01772004)) (14). He then had five consecutive declines in his calcitonin to 5,732 pg/ml and CEA levels remained overall stable at 22.0 ng/mL while on the immune checkpoint inhibitor avelumab, a > 40% decline not previously seen in his NCI clinical course (Figure 1A). Response assessment by RECIST v1.1 reported stable disease (Figure 2).

These findings coincided with an immune-related adverse event (asymptomatic rise in grade 3 lipase) that led to protocol-mandated treatment discontinuation. A subsequent analysis of the patient's lymph node resected post-vaccination revealed that the tumor was PD-L1 positive (Figure 1B). No baseline sample was available for evaluation given that the patient was diagnosed over 15 years prior to the latest surgery (15).

### Immune-Analysis

**Figure 3:**
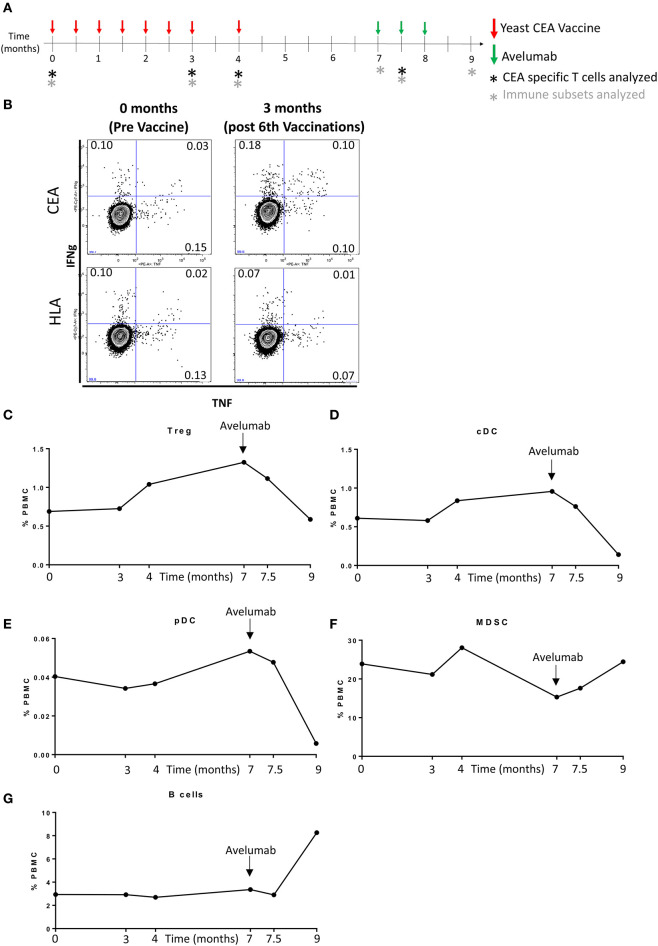
Induction of CEA-specific T cells and changes in peripheral immune cell subsets. **(A)** Schema showing the timing of sequential immunotherapies and immune assays. **(B)** CEA-specific T cells were identified in PBMCs by intracellular cytokine staining following a period of *in vitro* stimulation with overlapping 15-mer peptide pools encoding for the tumor-associated antigen CEA or the negative control peptide pool HLA. Dot plots of IFNγ and TNF production by CD4^+^ T cells showing induction of multifunctional CEA-specific T cells (producing >1 cytokine) at 3 months. **(C–G)** PBMCs were assessed for the frequency of 123 immune cell subsets over the course of immunotherapy. The most notable fluctuations were observed after initiation of avelumab (indicated by black arrow). The frequency over time of Tregs **(C)**, cDC **(D)**, pDC **(E)**, MDSC **(F)**, and B cells **(G)**, indicated as a percentage of total PBMCs.

Sufficient cryopreserved peripheral blood mononuclear cells (PBMCs) were available from this patient to analyze CEA-specific CD4^+^ and CD8^+^ T cell responses before vaccination, and after six and seven vaccinations with yeast-CEA, corresponding to 3 and 4 months, respectively; PBMCs were also examined 15 days following one cycle (administered every 2 weeks for 30 days) of avelumab (Figure 3A). This assay involves intracellular cytokine staining (ICS) following a period of *in vitro* stimulation (IVS) with overlapping 15-mer peptide pools encoding the tumor-associated antigen CEA or the negative control pool HLA, as previously described (16, 17). The patient did not have pre-existing CEA-specific T cells, but displayed a notable increase in CEA-specific T cells 3 months following yeast-CEA vaccination; following subtraction of background and any value obtained prior to vaccination, there were 488 CD4^+^ cells producing IFNγ and 438 CD4^+^ cells producing TNF per 1 × 10^6^ cells plated at the start of the stimulation assay. As visualized in the dot plots of Figure 3B, the CD4^+^ CEA-specific cells included multifunctional cells, or cells producing >1 cytokine. The increase in CEA-specific T cells was not seen at the two later time points evaluated.

The frequency of 123 PBMC subsets was also evaluated in this patient over his course of treatment at the National Cancer Institute using 11-color flow cytometry on cryopreserved PBMC as previously described (Supplemental Table 1) (18, 19). PBMCs were assayed prior to vaccination, 3 and 4 months following yeast-CEA vaccine, as well as at time points pre and post (15 and 42 days) avelumab (Figure 3A). Using 50% change as a cutoff, the first fluctuation in immune cell subsets was observed 4 months following vaccination with yeast-CEA, and included an increase in regulatory T-cells (Tregs) (51%), an inhibitory immune subset, compared to pre-vaccine levels (Figure 3C). After the patient completed vaccine and underwent surgery and prior to the initiation of avelumab, the patient had 92% more Tregs (Figure 3C) and 57% more conventional dendritic cells (cDC) (Figure 3D), a subset that is involved in antigen presentation, compared to pre-vaccine levels. The most dramatic fluctuations in immune subsets were noted at the time point after 6 weeks of avelumab, and included decreases in Tregs (Figure 3C), cDC (Figure 3D), and plasmacytoid DC (pDC, Figure 3E) compared to pre-avelumab therapy levels. pDC are tolerogenic DC, exhibiting poor immunostimulatory ability, and their interaction with T cells often favors the generation of Tregs (20). Increases in myeloid derived suppressor cells (MDSCs) (Figure 3F), another immune suppressive subset, and B cells (Figure 3G) were also noted after avelumab, compared to pre-avelumab levels. There were no alterations in the CD4^+^, CD8^+^, natural killer (NK) or NK-T compartments noted at any time point examined.

## Discussion

For many years, doxorubicin was the only US Food and Drug Administration (FDA)–approved treatment for patients with advanced thyroid cancer; however, response rate in patients with MTC is up to 20% with significant toxicity (21–23). Recently, in advanced MTC, several TKIs, such as axitinib, cabozantinib, gefitinib, lenvatinib, imatinib, motesanib, sorafenib, pazopanib, sunitinib, and vandetanib, have been studied in phase I, II, and III clinical trials. Vandetanib, an oral inhibitor of VEGFR (vascular endothelial growth factor receptor), RET, and EGFR (epidermal growth factor receptor) (24, 25) was approved by the FDA in April 2011 after a phase III trial demonstrated improved median progression-free survival (PFS) compared to placebo (hazard ratio 0.45, 95% CI 0.30–0.69) and overall response rate of 45% (26). Cabozantinib, an inhibitor of hepatocyte growth factor receptor (MET), VEGFR2, and RET, was approved by the FDA in 2012 after a phase III trial demonstrated improved median PFS of 11.2 months relative to 4 months in the placebo group (5, 27, 28). The impact of toxicity on patients was clearly indicated and for cabozantinib 70% of patients required dose reductions and 65% required dosing delays (27). Therefore, toxicity of FDA-approved TKI agents limits their use in patients with small volume, asymptomatic, or indolent disease (26). Furthermore, no clear data exist from these studies that either agent impacts overall survival. In addition, RET-specific TKIs in development are Selpercatinib (previously LOXO-292) and Blue-667 with more specific RET-targeting activity. These agents have demonstrated evidence of efficacy in early trial results (29, 30); however, further treatments are warranted with less toxicity.

Evidence for cell-mediated immunity to tumor-specific antigens has been found in medullary thyroid cancer (31) and early studies suggested that MTC-specific T cells exist (32, 33). Emerging data suggest that the immune system may be relevant in the treatment of MTC (34–36). Furthermore, immune-based treatments have been studied. Dentritic cell–based immunotherapy was given in patients with solid tumors, including MTC, and it was reported that vaccination with autologous tumor-pulsed DCs generated from peripheral blood was safe and can induce tumor-specific cellular cytotoxicity (9).

This case report may demonstrate the potential for therapeutic cancer vaccines to synergize with immune checkpoint inhibition sequentially in MTC and that principle could be applied as well to other cancers that may have tumor microenvironments (TMEs) devoid of baseline immune recognition. The therapeutic cancer vaccine in this trial was a heat-killed yeast-based vaccine designed to stimulate an immune response against CEA. After a phase I trial demonstrated safety (transient injection site reaction was the most common adverse event) and preliminary evidence of immunologic and clinical activity, a phase II study was developed in MTC (NCT01856920) (11). The phase I study included a patient with MTC who had substantial inflammation at sites of disease that followed 3 months of the vaccine (11). It is also possible that the patient's previous sunitinib is relevant in this case report. In a model using CEA-transgenic mice bearing CEA tumors, continuous sunitinib followed by vaccine increased intratumoral infiltration of antigen-specific T lymphocytes, decreased immunosuppressant Tregs and MDSCs, reduced tumor volumes and increased survival. The immunomodulatory activity of continuous sunitinib administration can create a more immune-permissive environment (37).

Despite the significant recent advances of anti-PD-1 and anti-PD-L1 therapy, they still impact only a minority of patients whose TMEs express those molecules at baseline. One hypothesis is that sequential use of vaccine can drive immune cells to the TME, resulting in an adaptive reaction by tumor cells (potentially from the presence of cytokines produced by active immune cells in the TME); upregulating PD-L1 and perhaps defining a role for anti-PD-L1/PD-1 therapies in patients who may not have otherwise benefited from such immunotherapies (38, 39). Based on this perspective, combining or sequencing vaccines with anti-PD-L1/PD-1 therapies could broaden the clinical benefit for all patients with immunologically “cold tumor microenvironments” (devoid of reactive immune cells) to enhance the clinical efficacy among cancer patients with a variety of tumor types. This case may provide an example of how increasing peripheral T-cell activation with vaccines can enable immune cells to then migrate to the TME and improve response rates to anti-PD-L1/PD-1 therapies (8, 13). Indeed, existing data with the FDA-approved therapeutic cancer vaccine for prostate cancer, sipuleucel-T, indicate that vaccine did increase T cells in the TME after treatment (40).

Induction of CEA-specific T cells was noted in the peripheral blood of this patient following vaccination with yeast-CEA, but not at later time points. It is possible the CEA-specific cells homed in on the TME inducing PD-L1 expression subsequently seen on the tumor. In addition, fluctuations in the peripheral immunome were noted in this patient over the course of therapy with yeast-CEA vaccine and subsequent avelumab therapy; these changes included both immune-potentiating and immune-suppressive alterations, with the most notable fluctuations occurring after several administrations of avelumab. The increase in suppressive elements may be a compensatory mechanism induced to tamper down the immune activation induced by the different immunotherapy treatments. However, as this patient had metastatic disease, it is unknown whether the changes in the peripheral immunome were directly induced by the sequential immunotherapy regimens or potentially related to disease progression.

As with all case reports, these presentations have limitations: the fact that the patient did not have a biopsy at baseline, prior to starting the vaccine, limits understanding of the baseline TME. Thus, it is unclear if the vaccine drove PD-L1 expression or if it was pre-existing in this patient. Little data exist for the presence of PD-L1 expression on MTC tumor cells. To further complicate this case's assessment, the patient was previously treated with sunitinib, which has been able to deplete Tregs, which alone or in combination with vaccine could have impacted the PD-L1 status of this patient (37). Nonetheless, data gleaned from using immunotherapy in such a rare disease are worth greater examination.

Although a decline in calcitonin does not directly correlate with clinical responses in this case or in MTC in general, the magnitude and consistency of the decline are noteworthy amidst data that suggest the predictive value of calcitonin doubling time and disease progression (41). Also, many patients with MTC have disease recurrence solely defined by serum tumor markers. In these patients, the opportunity to impact calcitonin kinetics with immunotherapy may decrease the pace of the disease and delay progression to overt metastasis requiring systemic therapies (TKIs) that are associated with toxicity or ultimately metastatic disease-related morbidity. Despite the effectiveness of TKIs in MTC, opportunities for immunotherapy clinical development may provide patients with additional treatment options that are less toxic and could thus be used earlier in the disease process.

## Ethics Statement

Written informed consent for publication of clinical details and/or clinical images was obtained from the patient.

## Author Contributions

JD, RD, and RM were responsible for study concept and design. JD and RD acquired the data from the study and prepared the manuscript. RD was responsible for the immune analysis and interpretation. RM reviewed the manuscript. JM, AG, MB, MR, LC, MM, WD, JS, and JG read and approved the final version of the manuscript. All authors contributed to the article and approved the submitted version.

## Conflict of Interest

The authors declare that the research was conducted in the absence of any commercial or financial relationships that could be construed as a potential conflict of interest.
