# Supplementary material for: A Case Report of Sequential Use of a Yeast-CEA Therapeutic Cancer Vaccine and Anti-PD-L1 Inhibitor in Metastatic Medullary Thyroid Cancer
Source: Front Endocrinol (Lausanne). 2020 Aug 7;11:490. doi: 10.3389/fendo.2020.00490 (PMC7427000; doi:10.3389/fendo.2020.00490)
Supplement: Supplementary file 1 [file Table_1.docx]

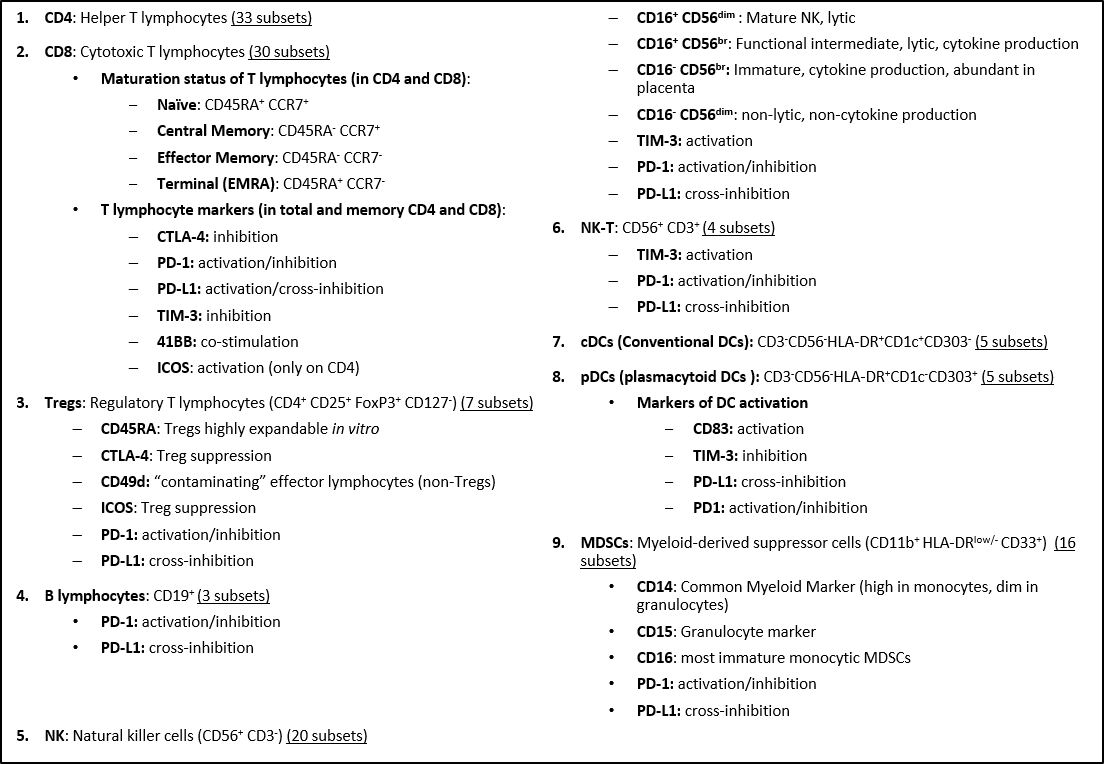


**Supplemental Table 1. Flow-cytometry analysis of 123 peripheral immune subsets.**

Subsets analyzed included 9 classic immune subsets and 114 refined subsets relating to the maturation/function of the classic immune cell types. Peripheral blood mononuclear cells (PBMCs) were monitored at various times pre- and post-therapy with yeast-CEA and subsequent avelumab therapy [Donahue et al (18)].
